# Improving the Oral Bioavailability of an Anti-Glioma Prodrug CAT3 Using Novel Solid Lipid Nanoparticles Containing Oleic Acid-CAT3 Conjugates

**DOI:** 10.3390/pharmaceutics12020126

**Published:** 2020-02-03

**Authors:** Hongliang Wang, Lin Li, Jun Ye, Rubing Wang, Renyun Wang, Jinping Hu, Yanan Wang, Wujun Dong, Xuejun Xia, Yanfang Yang, Yue Gao, Lili Gao, Yuling Liu

**Affiliations:** 1 State Key Laboratory of Bioactive Substance and Function of Natural Medicines, Institute of Materia Medica, Chinese Academy of Medical Sciences & Peking Union Medical College, Beijing 100050, China; wanghl@imm.ac.cn (H.W.);; 2 Beijing Key Laboratory of Drug Delivery Technology and Novel Formulation, Institute of Materia Medica, Chinese Academy of Medical Sciences & Peking Union Medical College, Beijing 100050, China

**Keywords:** phenanthroindolizidine, prodrug, oleic acid, conjugate, solid lipid nanoparticles, bioavailability

## Abstract

13a-(S)-3-pivaloyloxyl-6,7-dimethoxyphenanthro(9,10-b)-indolizidine (CAT3) is a novel oral anti-glioma pro-drug with a potent anti-tumor effect against temozolomide-resistant glioma in vivo. However, poor lipid solubility has limited the encapsulation efficacy during formulation development. Moreover, although the active metabolite of CAT3, 13a(S)-3-hydroxyl-6,7-dimethoxyphenanthro(9,10-b)-indolizidine (PF403), can penetrate the blood-brain barrier and approach the brain tissue with a 1000-fold higher anti-glioma activity than CAT3 in vitro, its bioavailability and *C*_max_ were considerably low in plasma, limiting the anti-tumor efficacy. In this study, a novel oleic acid-CAT3 conjugate (OA-CAT3) was synthesized at the first time to increase the lipid solubility of CAT3. The OA-CAT3 loaded solid lipid nanoparticles (OA-CAT3-SLN) were constructed using an ultrasonic technique to enhance the bioavailability and *C*_max_ of PF403 in plasma. Our results demonstrated that CAT3 was amorphous in the lipid core of OA-CAT3-SLN and the in vitro release was well controlled. Furthermore, the encapsulation efficacy and the zeta potential increased to 80.65 ± 6.79% and −26.7 ± 0.46 mV, respectively, compared to the normal CAT3 loaded SLN. As indicated by the high-performance liquid chromatography-mass spectrometry (HPLC-MS/MS) quantitation, the monolayer cellular transepithelial transport rate of OA-CAT3-SLN improved by 2.42-fold relied on cholesterol compared to the CAT3 suspension. Hence, the in vitro cell viability of OA-CAT3-SLN in C6 glioma cells decreased to 29.77% ± 2.13% and 10.75% ± 3.12% at 48 and 72 h, respectively. Finally, compared to the CAT3 suspension, the in vivo pharmacokinetics in rats indicated that the plasma bioavailability and *C*_max_ of PF403 as afforded by OA-CAT3-SLN increased by 1.7- and 5.5-fold, respectively. Overall, the results indicate that OA-CAT3-SLN could be an efficacious delivery system in the treatment of glioma.

## 1. Introduction

Glioblastoma multiforme (GBM) is an aggressive astrocytic cell neoplasm and is one of the leading causes of cancer-related deaths in both pediatric and adult populations, while also having poor clinical outcomes [1]. Notably, the median survival of patients with GBM is approximately 12–15 months after the initial diagnosis [2,3]. In 1999, the US Food and Drug Administration indicated oral temozolomide (TMZ), a DNA alkylating agent, as a first-line agent in the treatment of refractory anaplastic astrocytoma; in 2005, this indication was expanded to newly diagnosed glioblastoma patients [4,5]. However, temozolomide has been associated with severe toxicity, with the ensuing drug resistance limiting the efficacy and clinical use [6,7]. Therefore, it is necessary to further expand the development of novel therapeutic agents.

**Figure 1:**
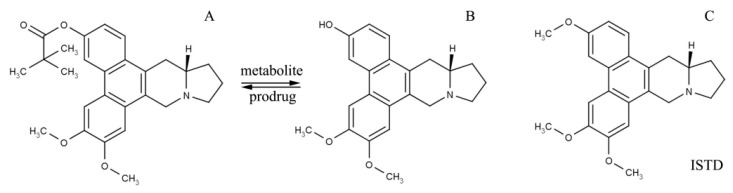
Molecular structure of CAT3 (**A**) as the pro-drug, PF403 (**B**) as the active in vivo metabolite, and (+)-Deoxytylophorinine (CAT) (**C**) as the internal standard (ISTD) of the drug concentration test in plasma.

CAT3 (Figure 1A) is a novel phenanthroindolizidine substance that selectively inhibits the hedgehog signaling pathway, demonstrating a significant inhibitory effect in GBM and TMZ-resistant GBM in orthotopic glioblastoma mice models following oral administration [8,9,10]. Moreover, central nervous system toxicity, one of the main obstacles in the development of phenanthroindolizidines, has not been associated with CAT3 [11]. PF403 (Figure 1B), reporting an in vitro anti-glioma activity approximately 1000 times higher than CAT3 [8] in human cell lines, is the active metabolite of CAT3 synthesized by esterases in vivo and can penetrate the blood-brain barrier to demonstrate an anti-glioma effect [12,13,14]. Although CAT3 can be absorbed via the gastrointestinal tract, it is a biopharmaceutics classification system class IV drug, insoluble in water, with poor bio-membrane permeability, and demonstrating low bioavailability and *C*_max_ of its active metabolite, PF403, in vivo. Moreover, the lipid solubility of CAT3 is also poor, further increasing the challenges associated with the development of appropriate lipid delivery vehicles. Hence, the poor bioavailability and the low aqueous and lipid solubilities remained crucial obstacles to the clinical application of CAT3.

Solid lipid nanoparticles (SLN) are interesting vectors for the oral delivery of lipophilic substances due to the excellent biocompatibility and assistance in drug uptake [15,16]. These nanoparticles can be prepared without the use of organic solvents and can be easily scaled up from laboratory to large-scale manufacture [17]. Moreover, as a lipid-based drug delivery system, SLN has been known to control drug release [18], promote oral absorption of drugs, modify the pharmacokinetics and pharmacodynamics [19,20], enhance tissue or cell-specific targeting [21], adjust tissue distribution [20], and reduce side effects [22,23,24]. Hence, SLN could be a superior drug delivery system to load CAT3 and improve bioavailability.

However, it has been reported that the lipid core of SLN demonstrates a perfect crystal lattice and cannot contain an excess amount of drug molecules [25]. During the encapsulation of CAT3 in SLN, the same phenomenon was observed, and the drug loading (DL) and the encapsulation efficiency (EE) were extremely low. Previously, studies have reported that some type of liquid lipid, for example, low melting point fatty acids or oils, could be used to formulate a less-ordered crystalline structure of the lipid core, increasing the DL and EE [26]. To construct the CAT3 loaded SLN, several liquid lipids were tested and oleic acid (OA) was selected as an ideal agent. OA is a commonly used lipid to prepare SLN [27,28], with its carboxyl group forming an ionic bond with the CAT3 nitrogen atom, resulting in a conjugate that improves lipid solubility. Moreover, even with identical compositions and contents in the SLN, the results obtained by the different methods of preparation differed significantly. The product obtained by conjugating OA and CAT3 (OA-CAT3) first, and preparing the SLN next demonstrated a higher solubility in lipids, with a higher DL and EE than the normal SLN, prepared by simply mixing together CAT3 and OA. Hence, OA-CAT3 may be a key complex contributing to the preparation of the SLN.

This study aimed to formulate a novel OA-CAT3 by linking OA and CAT3 through ionic bonding to overcome the challenges of CAT3 insolubility in lipids, forming the OA-CAT3 loaded SLN (OA-CAT3-SLN) using a hot ultra-sonication technique. Simultaneously, normal CAT3 loaded SLN (CAT3-SLN, with the same composition and contents as OA-CAT3-SLN except for the advanced preparation of OA-CAT3) were prepared using the same technique to explore the OA-CAT3 mechanism promoting the encapsulation of CAT3 by SLN. Furthermore, we evaluated the extent of bioavailability improvement in the active metabolite, PF403, by OA-CAT3-SLN. To achieve these goals, the size distribution, zeta potential, DL, EE, solid-state characterization of the drug, and in vitro release were compared between OA-CAT3-SLN and CAT3-SLN. Furthermore, the Caco-2 cellular uptake, Madin-Darby canine kidney cells transfected with the human MDR1 gene (MDCK-MDR1) monolayer transfer, and the C6 glioma cell cytotoxic activity of OA-CAT3-SLN were evaluated in vitro. Finally, an in vivo pharmacokinetic evaluation of OA-CAT3-SLN was conducted in Sprague-Dawley (SD) rats using the CAT3 suspension, OA-CAT3, and CAT3-SLN as controls.

## 2. Materials and Methods

### 2.1. Materials

CAT3 (purity > 99%), PF403 (purity > 99%), and (+)-Deoxytylophorinine (CAT) (purity > 99%) were prepared at our institute (Institute of Materia Medica, Chinese Academy of Medical Sciences & Peking Union Medical College, Beijing, China), and the structures were confirmed by infrared, two-dimensional nuclear magnetic resonance, and Fourier transform mass spectrometry. OA and Lipoid S75 (soy lecithin with 68% phosphatidylcholine) were both purchased from Lipoid KG (Ludwigshafen, Germany). Poloxamer188 (Pluronic F68) and Tween 80 were received as gifts from BASF (Ludwigshafen, Germany). Compritol 888 ATO was received as a gift from Gattefosse (Saint-Priest Cedex, France). The solvents for high-performance liquid chromatography (HPLC) were obtained from Thermo Fisher Scientific (Waltham, MA, USA). All other chemicals used were of analytical grade.

#### 2.1.1. Cell Culture

MDCK-MDR1 cells expressing a high level of P-gp were generously provided by Professor Li (Institute of Materia Medica, Chinese Academy of Medical Sciences & Peking Union Medical College, Beijing, China). Luciferase-expressing C6 glioma cells (C6-luc) were generously provided by Professor Huang (Institute of Materia Medica, Chinese Academy of Medical Sciences & Peking Union Medical College, Beijing, China). Caco-2 cells were purchased from the Cell Resource Center, Peking Union Medical College (Beijing, China). MDCK-MDR1, C6, and Caco-2 cells were cultured in Dulbecco’s Modified Eagle’s Medium (Hyclone, Logan, UT, USA) supplemented with 10% fetal bovine serum (Gibco, Thermo Fisher Scientific) and 1% penicillin/streptomycin (Gibco, Thermo Fisher Scientific) under 5% CO_2_ at 37 °C.

#### 2.1.2. Animals

Sprague Dawley rats (males, 200–250 g) were purchased from Beijing Vital River Laboratory Animal Technology (Beijing, China), and were raised at the Institute of Material Medica, the Chinese Academy of Medical Sciences, and the Peking Union Medical College (Beijing, China). The experiments were performed under the approval of the Laboratory Animal Care and Use Committee of the Peking Union Medical College on 11 April 2017 and the animal experiment lasted to 25 November 2017 (project identification code 00005964). All animal experiments were performed in accordance with the guidelines of laboratory animals-guidelines for ethical review of animal welfare (GB_T 35892.2018).

### 2.2. Methods

#### 2.2.1. Preparation and Spectral Analysis of OA-CAT3

OA-CAT3 was prepared by the addition of CAT3 into the excess OA (the molar ratio was 1:7) and by maintaining the mixture in the ultrasonic cleaning machine at 45 °C for 30 min. After an additional 10 h at 45 °C, the OA-CAT3 was obtained as a clear yellowish oil, which was used to prepare the OA-CAT3-SLN. The reaction time and temperature were optimized using nuclear magnetic resonance (NMR), differential scanning calorimetry (DSC), and Fourier transform infrared spectroscopy (FTIR). In addition, 45 °C was selected to avoid the darkening of OA due to the oxidation by the air at higher temperatures.

To reduce the interference of excess OA in the results, the OA-CAT3 sample was prepared at a molar ratio of 1:1 and analyzed by ^1^H NMR, FTIR, and DSC. Briefly, equimolar CAT3 and OA was added to a solution of acetonitrile and the mixture was heated under reflux for 2 h. After concentration under reduced pressure, the residue was washed with diethyl ether and then concentrated to afford OA-CAT3.

^1^H NMR (600 MHz) spectra were recorded in dimethyl sulfoxide (DMSO) on a Bruker-Avance 600 spectrometer at 303 K and with tetramethylsilane (TMS) as an internal standard (chemical shifts (δ) in parts per million, coupling constants (J) in hertz).

CAT3 FTIR spectra were obtained using an FTIR spectrometer (Nicolet 5700 FTIR Spectrometer, Thermo Fisher Scientific, Waltham, MA, USA). CAT3 (equivalent to 2 mg) was mixed with KBr (about 30 mg) and ground into a fine powder using an agate mortar, before compressing into the KBr disk under a press at 10,000 psi. The baseline was corrected, and the samples were scanned against a blank KBr pellet background at a wave number ranging between 4000 and 400 cm^−1^ with a resolution of 4.0 cm^−1^, with 64 scans. Next, the characteristic peaks were recorded. To test the OA-CAT3 and the OA, the same instrument equipped with an infrared microscope (Nicolet Centaur μs, Thermo Fisher Scientific) was used.

DSC 6200 (Seiko Instruments, Tokyo, Japan) was used to obtain the thermograms of CAT3, OA, and OA-CAT3. The indium (156.6 ± 0.2 °C) and zinc (419.5 ± 0.3 °C) standards were used to calibrate the melting point of the apparatus. The heat flow and enthalpy of the apparatus were calibrated by the indium heat of fusion (28.58 ± 0.3 J·g^−1^). The samples were placed in the standard aluminum sample pans and an empty pan was used as a reference. The samples were tested at between the temperature range of −20 and 250 °C at the heating rate of 10 °C/min for OA and OA-CAT3, and the range of 28 and 280 °C for CAT3, as it is a solid drug at 25 °C and there was no need to start the test from an extremely low temperature. Nitrogen was used as the gas purged at a flow rate of 60 mL/min.

#### 2.2.2. Preparation of OA-CAT3-SLN and CAT3-SLN

**Figure 2:**
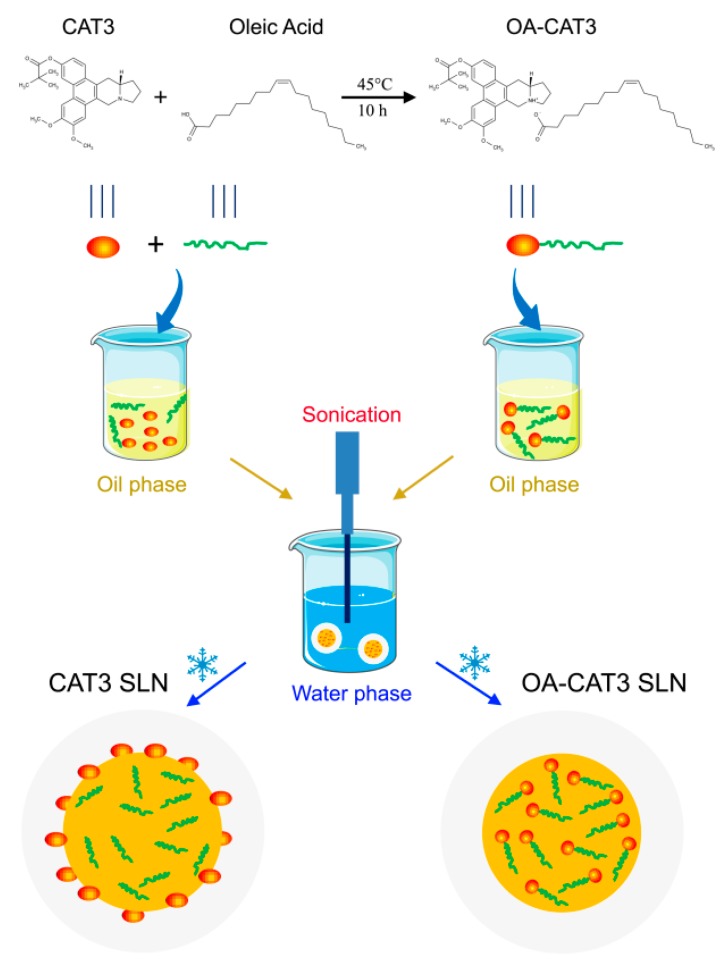
Main preparation process of OA-CAT3-SLN and CAT3-SLN.

The main preparation processes of OA-CAT3-SLN and CAT3-SLN are shown in Figure 2. Both formulations were prepared using hot homogenization followed by the hot ultra-sonication method [29,30]. To prepare the OA-CAT3-SLN, compritol 888 ATO 0.3 g and Lipoid S75 0.2 g were heated to 80 °C and dissolved as the oil phase. Next, approximately 60 mg of OA-CAT3 (containing 10 mg of CAT3) was dispersed in the oil phase. Additionally, 9.3 mL aqueous solution of surfactant (containing 0.1 g each of Tween 80 and Poloxamer188) was heated to the same temperature and referred to as aqueous phase. The aqueous phase and oil phase were then mixed sufficiently using the homogenizer (FA25, Fluko Equipment, Shanghai, China) at 16,000 rpm for about 3 min in a water bath at 80 °C. The obtained dispersion was sonicated with the help of a “Probe Sonicator” (Sonic prepultra homogenizer, Osterode, Germany) for 8 min at 300 Watt in an 80 °C water bath. Then, the OA-CAT3-SLN suspension was obtained after cooling in an ice bath, sealed in a glass vial, and stored at 4 °C. Similarly, the CAT3-SLN was prepared by the same method except for the solution of 10 mg CAT3 that was dissolved in 50 mg OA directly and then added into the oil phase without prior formation of OA-CAT3. The blank SLN was prepared using the same method with the absence of CAT3. The amount of all ingredients and the preparation parameters were optimized with the minimum particle size, and the maximum DL and EE to ensure the quality of SLN products.

#### 2.2.3. Physicochemical Characterization of OA-CAT3-SLN and CAT3-SLN

##### Morphology and Particle Size

The morphological evaluation of OA-CAT3-SLN was carried out by transmission electron microscopy (TEM, JEM-1400 plus; JEOL, Tokyo, Japan). The OA-CAT3-SLN was diluted with deionized (DI) water at a ratio of 1:100 and mixed by slight shaking. One drop of the diluted sample was deposited on a film-coated copper grid and negatively stained with 2% (w/v) phosphotungstic acid before observation under the electron microscope.

The average particle size, polydispersity index (PDI), and zeta potential of the prepared SLN were measured by photon correlation spectroscopy using a Malvern Zetasizer Nano ZS90 (Malvern Instruments, Malvern, UK). The samples were diluted with DI water (1:100) and the measurements were performed at 25 °C. The scattering angle for measurement was 90°. To measure of particle size and PDI, the samples were placed in disposable polystyrene cells. For the zeta potential measurements, the samples were placed in disposable plain folded capillary zeta cells. All experiments were performed in triplicates and the data were analyzed using built-in software. The results are represented as the mean ± standard deviation.

##### Differential Scanning Calorimetry (DSC) and Powder X-ray Diffractometry (PXRD)

Differential scanning calorimetry (DSC) and powder X-ray diffractometry (PXRD) were used to identify the crystal stage of CAT3 present in the OA-CAT3-SLN or CAT3-SLN. As both DSC and PXRD required a solid sample for measurement, SLN samples (including OA-CAT3-SLN, CAT3-SLN, and the blank SLN) were subjected to lyophilization for 48 h using a freeze-drying apparatus (Alpha 1-4, Martin Christ, Osterode, Germany) under standard conditions.

The DSC analyses of OA-CAT3-SLN, CAT3-SLN, blank SLN, CAT3, and the physical mixture of blank SLN and the CAT3 were performed in the temperature range of 28–280 °C, with a heating rate of 10 °C/min.

The X-ray diffraction patterns of lyophilized OA-CAT3-SLN, CAT3-SLN, blank SLN, CAT3, and the physical mixture of blank SLN and the CAT3 were studied using a powder X-ray diffractometer (D8 Advance-Bruker, Billerica, MA, USA). PXRD studies were performed by exposing the samples to Cu Kα radiation (40 kV, 30 mA) and scanned from 2–60°, 2θ at a step size of 0.045° and step time of 0.5 s [30,31].

##### Drug Loading (DL) and Encapsulation Efficiency (EE)

The DL and EE of CAT3 incorporated in OA-CAT3-SLN or CAT3-SLN were determined by spectrophotometric analysis using a UV-visible spectrophotometer HP 8453 (Agilent Technologies, Santa Clara, CA, USA) at 263 nm. Prior to the total CAT3 content analysis, OA-CAT3-SLN and CAT3-SLN were dissolved in ethanol at 50 °C and filtered using a 0.22 µm polyvinylidene fluoride (PVDF) syringe filter.

The efficiency of CAT3 entrapped in SLN was calculated using the ultracentrifugation method using centrifugal devices (Amicon^®^ Ultra-4 100k, Millipore, Billerica, MA, USA) with a cut-off membrane of 100 kDa [32]. Briefly, SLN (1 mL) were placed in the ultrafiltration tubes and centrifuged in a high-speed Dragon D3024R Lab cooling centrifuge (Dragon Laboratory Instruments, Beijing, China) at 15,000 *g* for 30 min at 4 °C. The amount of free CAT3 in the ultra-filtrate solution was analyzed by spectrophotometric analysis.

The DL and EE (%) of SLN were estimated by using the following equations.

(1) $$\mathrm{Drug}\;\mathrm{Loading}\;\left(\%\right)=\frac{\mathrm{amount}\;\mathrm{of}\;\mathrm{total}\;\mathrm{CAT}3\;-\;\mathrm{free}\;\mathrm{CAT}3}{\mathrm{amount}\;\mathrm{of}\;\mathrm{carriers}\;+\;\mathrm{total}\;\mathrm{CAT}3\;-\;\mathrm{free}\;\mathrm{CAT}3}$$

(2) $$\mathrm{Encapsulation}\;\mathrm{Efficiency}\;\left(\%\right)=\;\frac{\mathrm{amount}\;\mathrm{of}\;\mathrm{total}\;\mathrm{CAT}3-\;\mathrm{free}\;\mathrm{CAT}3}{\mathrm{amount}\;\mathrm{of}\;\mathrm{total}\;\mathrm{CAT}3}\times 100$$

##### In Vitro Release and Releasing Mechanism

Drug release from SLN was monitored in vitro under simulated gastrointestinal conditions. The tests were performed by placing 2 mL SLN samples (containing 2 mg CAT3) into 400 mL of the dissolution medium maintained at 37 °C. The dissolution media contained 0.1% SDS (w/v) to meet sink conditions. Preswollen 8–10 kDa MWCO dialysis bags (Sigma-Aldrich, Madrid, Spain), which contained 5 mL of the blank SDS solution with the same concentration, were placed into the dissolution flask (*n* = 3). The release behavior of CAT3 was compared under the same condition by placing 5 mg CAT3 powder into the 1000 mL media. The samples (1 mL) were withdrawn at predetermined time points from the dialysis bags and replaced with the same amount of fresh medium maintained at 37 °C. The samples were centrifuged for 5 min at 12,000 rpm, and the resultant supernatants were quantified for drug content. The samples were protected from light during the experiments. The DDsolver software [33,34] was used to evaluate the drug-release kinetics. An Excel worksheet (Microsoft Corporation, Redmond, WA, USA) was used to control the computing method for comparison with the results obtained using the DDSolver program. According to the example format for each built-in module of the DDSolver, all data were entered into Excel. The relevant parameters were calculated following the step-by-step equations utilizing an Excel spreadsheet.

#### 2.2.4. In Vitro and In Vivo Bio Evaluation of OA-CAT3-SLN

##### Transepithelial Transport Study

The permeabilities of CAT3 and OA-CAT3-SLN were investigated on an MDCK-MDR1 cell monolayer. MDCK-MDR1 cells were seeded onto 12-well transwell^®^ filter inserts (pore size 0.4 μm, surface area 1.12 cm^2^; Corning Incorporated, Corning, NY, USA) at a density of 2 × 10^5^ cells/well. The culture medium was changed every 24 h for seven days, and the cell monolayers with a transepithelial electrical resistance (TEER) above 110 Ω·cm^2^ were used for the transport experiments. Prior to performing the TEER test, the culture medium was removed, and the monolayer was pre-incubated with 0.5 mL of Hanks’ balanced salt solution (HBSS) for 20 min at 37 °C.

After measuring TEER, HBSS was removed, and 0.5 mL of 100 ng/mL CAT3 or the same concentration of OA-CAT3-SLN diluted with HBSS was added to the apical side (AP), and 1.5 mL of HBSS was added to the basolateral (BL) compartment. The monolayer was incubated for 2 h at 37 °C. Samples (50 μL) were obtained from the BL compartment at 0.5, 1, 1.5, and 2 h. Next, the samples were stored at 4 °C for analysis.

To investigate the mechanism of cellular internalization of OA-CAT3-SLN, various specific endocytic inhibitors were evaluated in the uptake study. Chlorpromazine (10 μg/mL) was used as the clathrin-mediated endocytic inhibitor, nystatin (5 μg/mL) as the caveolae structure and function abolisher, and methyl-β-Cyclodextrin (MCYP, 13 mg/mL) as the disruptor of lipid rafts [35,36,37]. The inhibitors were added to the cell monolayer and pre-treated for 30 min before the test. After the treatments, the cells were treated with OA-CAT3-SLN for a further 2 h using the same method. To investigate whether the GTPase dynamin played an important role in endocytosis, the permeability of OA-CAT3-SLN was investigated at 4 °C.

The amount of CAT3 or OA-CAT3-SLN that permeated that through the monolayer was determined using the liquid chromatography-mass spectrometry (LC-MS/MS) system. The apparent permeability coefficient (*P*_app_) of CAT3 or OA-CAT3-SLN was calculated according to the following equation: *P*_app_ = d*Q*/d*t* × 1/(*A* × *C*_0_), where d*Q*/d*t* indicates the linear appearance rate of mass at the BL side (μmol/s), *C*_0_ is the initial concentration of CAT3 or OA-CAT3-SLN on the AP side (μmol/mL), and *A* is the surface area of the monolayer (cm^2^).

##### In vitro Cellular Uptake Study

To verify whether SLN can enhance intracellular delivery, a fluorescence microscopic study was performed on the Caco-2 cells and C6 cells. Fluorescent coumarin 6 (Cou-6) was loaded in the blank SLN [38]. Caco-2 and C6 cells were seeded in six-well plates containing a cover glass seeded with 2 × 10^5^ cells/well in the cell culture medium overnight. Cou-6 loaded SLN or the Cou-6 solution in 2 mL DMEM medium (Cou-6 concentration: 100 ng/mL) was added and incubated at 37 °C for 1 h. Simultaneously, another Cou-6 loaded SLN was incubated at 4 °C for 1 h. To validate the cellular uptake through lipid rafts, 13 mg/mL MCYP was added into another well containing Caco-2 cells and was pre-treated for 30 min before conducting the test at 37 °C. After incubation with the Cou-6, the cells were washed three times with cold phosphate-buffered saline (PBS) and fixed with 4% paraformaldehyde for 15 min. Next, the cells were washed three times with cold PBS and stained with 4′,6-diamidino-2-phenylindole (DAPI). Later, the coverslips were visualized using a laser scanning confocal microscope (LSCM, Leica TCS SP8 X, Wetzlar, Germany) using appropriate excitation and emission wavelengths (405/450 nm for DAPI and 470/504 nm for Cou-6) [38,39].

##### In Vitro Cytotoxicity Analysis

Cytotoxicity of the CAT3 suspension, OA-CAT3-SLN, and blank SLN against the C6 brain glioma cell line was measured by 3-(4,5-dimethylthiazol-2-yl)-2,5 diphenyltetrazolium bromide (MTT) assay. Briefly, the cells were seeded into 96-well plates at a cell density of 2 × 10^4^ cells/mL (100 μL/well). After 24 h of incubation, the cells were further incubated for 24, 48, and 72 h in fresh DMEM complete medium containing 0.64 ng/mL CAT3 suspension, OA-CAT3-SLN, or the blank SLN, respectively. At the above-mentioned time points, the culture medium was replaced by the MTT solution (0.5 mg/mL, 200 μL/well), followed by incubation for further 4 h. The supernatant was removed and 100 μL of DMSO solution was added to the wells. The absorbance values of each well were measured at 570 nm using a microplate reader (Synergy H1 Hybrid Multi-Mode Microplate Reader, BioTek, Winooski, VT, USA).

##### In Vivo Bioavailability Studies

For in vivo analysis of CAT3 and its metabolite, PF403, in the rat plasma, 10 μL of the internal standard (ISTD) and ((+)-Deoxytylophorinine, CAT, 10 ng/mL) was added to 50 μL of plasma. After the addition of 90 μL of acetonitrile for the precipitation of protein, the sample was vortex-mixed for 30 s, followed by centrifugation at 13,000 rpm for 10 min. The supernatant was transferred to a new tube, and a 5-μL aliquot of the solution was injected into the LC-MS/MS for analysis.

Triple-quadrupole mass spectroscopy (6410B, Agilent, Santa Clara, CA, USA) was performed under multiple reaction monitoring and a positive electrospray ionization mode. Nitrogen (350 °C) as the nebulizer gas was flown at 10 L/min and the pressure was set to 40 psi. The voltages of the fragmentor potential and collision energy were 135 and 20 eV, respectively. The ion reactions for the determination of CAT3, PF403, and ISTD were *m/z* 434.3→*m/z* 70.1, 350.2→70.1, and 364.2→70.1, respectively.

The analyte concentrations were determined using the MassHunter software (Agilent). Chromatographic separation was performed on a 1200 serious RRLC system (Agilent) with a C_18_ column (50 mm by 2.1 mm, 1.8 μm, Agilent) and a corresponding guard column (ODS, 5 μm). DI water and 0.1% formic acid acetonitrile (20:80, *v/v*) were used as the mobile phase for elution. The flow rate was 0.4 mL/min at 40 °C.

The primary pharmacokinetic parameters were calculated based on the statistical moment method using Drug and Statistics (DAS) 3.0 software (BioGuider Medicinal Technology Co. Ltd., Shanghai, China). Parameters included: Peak concentration (*C*_max_), time of peak concentration (*T*_max_), area under the concentration-time curve from 0 h to *t* (AUC_0–*t*_), area under the concentration-time curve from 0 h to ∞ (AUC_0–∞_), average residence time from 0 h to *t* (MRT_0–*t*_), average residence time from 0 h to ∞ (MRT_0–∞_), and elimination half-life of the drug concentration (*t*_1/2z_). The non-compartmental model was used to estimate the pharmacokinetic parameters.

For pharmacokinetic studies, 24 rats were divided into four groups (*n* = 6 per group). Prior to the day of administration, the rats were fasted for 12 h, allowing water ad libitum. OA-CAT3-SLN (10 mg/kg) or the same dose of the CAT3 suspension (dispersed in 0.5% CMC-Na solution), OA-CAT3, and CAT3-SLN was administered orally and the drug content was pre-analyzed by spectrophotometric methods at 263 nm to ensure dose accuracy. Blood samples (about 150 μL) were collected into heparinized tubes from each rat through a retro-orbital sinus puncture that was performed at 0, 5, 10, 15, and 30 min and 1, 2, 4, 6, 8, 12, and 24 h after oral administration. The blood samples were immediately processed to obtain the plasma by centrifuging at 4000 rpm for 10 min. Plasma was collected and frozen at −80 °C until analysis by LC-MS/MS.

#### 2.2.5. Statistical Analysis

The statistical difference between the treatments was evaluated using the Analysis of Variance (ANOVA) test. Data are reported as mean ± standard deviation (SD). Statistical significance of differences was evaluated by Duncan’s multiple range test (*p* ≤ 0.05) using SPSS software version 17.0 (SPSS Inc., Chicago, IL, USA).

## 3. Results

### 3.1. Characterization of OA-CAT3 by NMR, Fourier Transform Infrared Spectroscopy (FTIR), and Differential Scanning Calorimetry (DSC)

**Figure 3:**
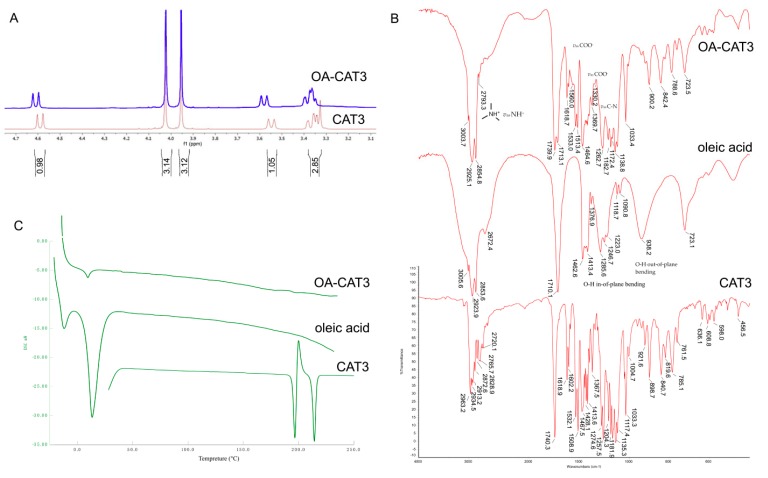
^1^H NMR (**A**), Fourier transform infrared spectroscopy (FTIR) spectra (**B**), and differential scanning calorimetry (DSC) thermograms (**C**) of CAT3, oleic acid (OA), and oleic acid and CAT3 conjugate (OA-CAT3).

Figure 2 shows the reaction method between the CAT3 and OA. Figure 3A shows the ^1^H NMR spectra comparison of CAT3 and OA-CAT3. The chemical shifts at 3.4, 3.6, and 4.6 ppm were assigned to the five protons beside the nitrogen atom of CAT3. When the nitrogen atom combined with the hydrogen ion of carboxyl in OA, their chemical shifts moved to the lower field, indicating the formation of a conjugate linkage.

The successful conjugation of OA-CAT3 was also proved by FTIR analysis. The FTIR spectra of CAT3, OA, and OA-CAT3 are presented in Figure 3B. The band at 2793 cm^−1^ of OA-CAT3 can be referred to NH^+^ asymmetric stretching (*v*_as_ NH^+^). Furthermore, the bands at 1560 cm^−1^ and 1330 cm^−1^ of OA-CAT3 can be referred to COO^–^ asymmetric stretching (*v*_as_ COO^−^). Next, the absorption band at 1172 cm^−1^ of OA-CAT3 was attributed to *v*_as_ C–N. Finally, the prominent bands at 1413 cm^−1^ and 938 cm^−1^ were assigned to the –OH in-of-plane and out-of-plane bending of the carboxyl group of OA, respectively. These bands disappeared from the spectra for OA-CAT3, indicating the formation of the conjugate.

DSC thermograms of CAT3, OA, and OA-CAT3 conjugate are shown in Figure 3C. The DSC thermograms of pure OA indicated two endothermic peaks at −11.8 and 13.7 °C, respectively, corresponding to the crystal transformation from the γ-form to α-form and the intrinsic melting point of α-form [28]. CAT3 crystallinity decreased due to the molecular interaction between CAT3 and OA.

### 3.2. Characterizations of OA-CAT3-SLN and CAT3-SLN

#### 3.2.1. Droplet Size, PDI, Zeta Potential, and Morphology

**Figure 4:**
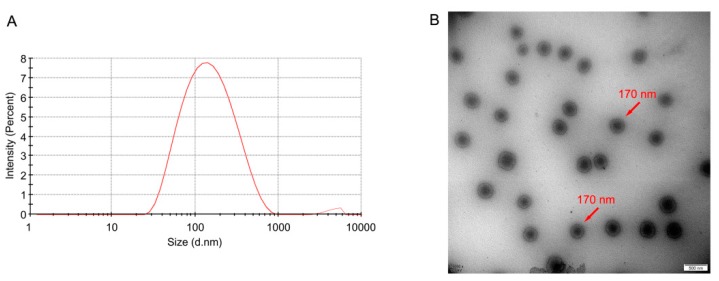
Particle size distribution (**A**) and transmission electron microscopy (TEM) image (**B**) of OA-CAT3 loaded solid lipid nanoparticles (SLN) (OA-CAT3-SLN). (Scale bar: 200 nm).

Particle size is one of the most important factors of SLN [40,41,42,43]. As shown in Table 1, the average particle sizes of the prepared SLN formulations were 139.2 ± 2.47, 151.3 ± 17.51, and 155.7 ± 3.03 nm for blank SLN, OA-CAT3-SLN, and CAT3-SLN, respectively. The PDI of all SLN formulations were less than 0.3, which indicates that the preparations had an ideal and narrow distribution and followed unimodal normal distribution (Figure 4A) [44].

The TEM image of OA-CAT3-SLN is shown in Figure 4B, which demonstrate that SLN are spherical in shape with an average size of about 170 nm.

The zeta potential indicates the degree of repulsion between similarly charged, adjacent particles in dispersion, and a higher zeta potential will confer stability and prevent aggregation [45]. Table 1 shows the mean zeta potential of SLN. The zeta potential values of blank SLN, OA-CAT3-SLN, and CAT3-SLN were −41.7 ± 0.80 mV, −26.7 ± 0.46 mV, and −7.9 ± 0.15 mV, respectively.

#### 3.2.2. Drug Loading (DL) and Encapsulation Efficiency (EE)

The DL and EE were determined by spectrophotometric analysis at 263 nm. Linear range, repeatability, and recovery were calculated under optimal conditions. To estimate the repeatability, shown as relative standard deviation percentage (%RSD), six replicates were employed and the %RSD value was found to be less than 1.0%. Satisfactory recoveries (99.8%) and low RSD (less than 2.0%) were achieved. Good linearity was obtained (*r*^2^ > 0.9999) in the concentration range of 1–8 μg/mL for CAT3 (Formula (3)). In the formula, *c* represents the concentration of CAT3 as μg/mL.

(3) Absorbance=0.1118×c+0.0063

Table 1 shows the influence of OA-CAT3 on EE and DL. The EE of OA-CAT3-SLN (80.65 ± 6.79%) was significantly higher than that of CAT3-SLN (58.48 ± 3.35%). A statistically significant difference in the DL of OA-CAT3-SLN was observed compared to that of CAT3-SLN. The DL was 5.478 ± 0.346% for OA-CAT3-SLN and 4.067 ± 0.163% for CAT3-SLN.

#### 3.2.3. In Vitro Controlled Release Study

**Figure 5:**
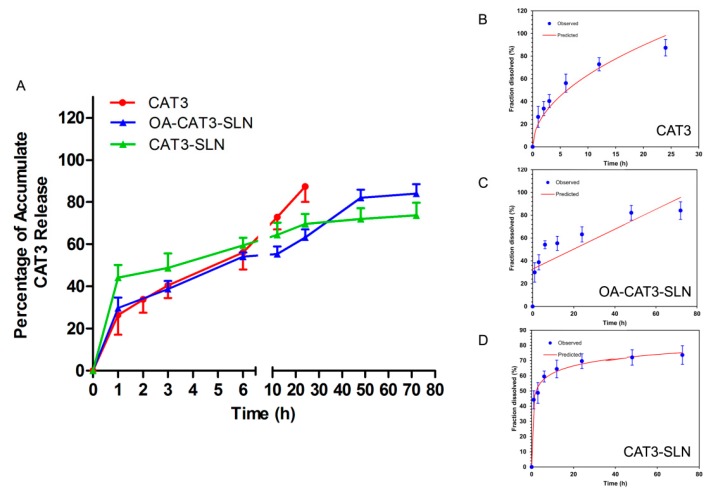
In vitro accumulate drug release percentage versus time of CAT3, OA-CAT3-SLN, and CAT3-SLN (**A**), Higuchi model of CAT3 suspension release (**B**), zero-order with the F0 model of OA-CAT3-SLN release (**C**), and Weibull model of CAT3-SLN release (**D**).

Figure 5A shows the accumulative CAT3 release in the dissolution media. In the first 24 h, more than 80% of CAT3 was released from the CAT3 suspension, conforming to the Higuchi model equation (Formula (4) and Figure 5B). Both OA-CAT3-SLN and CAT3-SLN displayed biphasic drug release patterns, with burst release at the initial stage and prolonged release later. However, OA-CAT3-SLN (29.84 ± 8.53%) exhibited a lower burst release than CAT3-SLN (44.1 ± 5.97%) and can release completely (84.08 ± 7.76%) versus CAT3-SLN (73.65 ± 6.14%) at 72 h. The lower burst drug release at the initial stage and better release control [46,47] at 72 h can be achieved by introducing OA-CAT3 conjugates into the formulation. The release profile of OA-CAT3-SLN and CAT3-SLN conformed to the zero-order with the F0 model equation (Formula (5) and Figure 5C) and Weibull model equation (Formula (6) and Figure 5D), respectively. In the formulas, *F* represents the fraction of drug released in time *t*.

(4) $$F=20.044\;\times t^{1/2}$$

(5) F=32.833+0.827×t

(6) $$F=100\times \left(1-e^{-{\left(t-0.292\right)}^{\frac{0.191}{1.633}}}\right)$$

#### 3.2.4. Differential Scanning Calorimetry (DSC) and Powder X-ray Diffractometry (PXRD) Studies

**Figure 6:**
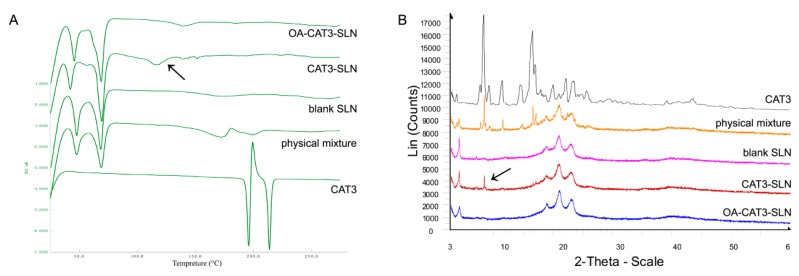
DSC thermograms (**A**) and powder X-ray diffractometry (PXRD) patterns (**B**) of lyophilized OA-CAT3-SLN, CAT3-SLN, blank SLN, CAT3, and the physical mixture of blank SLN with CAT3.

DSC thermograms of CAT3, the physical mixture of CAT3 with blank-SLN, blank-SLN, CAT3-SLN, and OA-CAT3-SLN are shown in Figure 6A. There are two sharp melting endothermic peaks at 195.8 and 213.7 °C with an enthalpy of 38.5 and 24.6 J·g^−1^, respectively, in the CAT3 sample, indicating that the CAT3 was a mixture of two crystal forms. The melting endotherm of blank SLN ranged from 40 to 75 °C, which indicated that the core of the blank SLN was crystalline and solid. The characteristic melting endotherm of CAT3 was observed in the physical mixture samples of the blank SLN and the CAT3, which were 172.0 and 199.1 °C, respectively. From the DSC curve of OA-CAT3-SLN, the melting endotherm of CAT3 was not observed, implying that the CAT3 was present in an amorphous state. However, the melting endotherms were at 145.6 and 170.8 °C in the CAT3-SLN, implying that CAT3 was still partially crystalline in CAT3-SLN.

The PXRD results are shown in Figure 6B. CAT3 was a crystalline substance, with *d* values of 10.663, 5.363, 5.217, 4.002, 7.748, 3.778, 6.094, and 11.487. There were wide diffraction peaks at *d* values of 4.229, 21.191, 3.865, and 4.689 in blank SLN, indicating that its lipid core was crystalline. The crystallographic diffraction peaks of blank SLN and CAT3 physical mixture were a simple superposition of the CAT3 and the blank SLN diffraction peaks. The crystalline peaks of CAT3 were absent in the OA-CAT3-SLN sample, indicating that the drugs were in amorphous form. However, the main characteristic diffraction peak of CAT3 at *d* values of 10.663, 5.363, and 5.217 was found in the CAT3-SLN sample, indicating CAT3 was still in the crystalline form. Consistent with the DSC results, the results of PXRD proved that OA-CAT3 was necessary for the composition of CAT3 loaded SLN to obtain an amorphous drug.

### 3.3. In Vitro and In Vivo Bio Evaluation of OA-CAT3-SLN

#### 3.3.1. Transepithelial Transport Study

**Figure 7:**
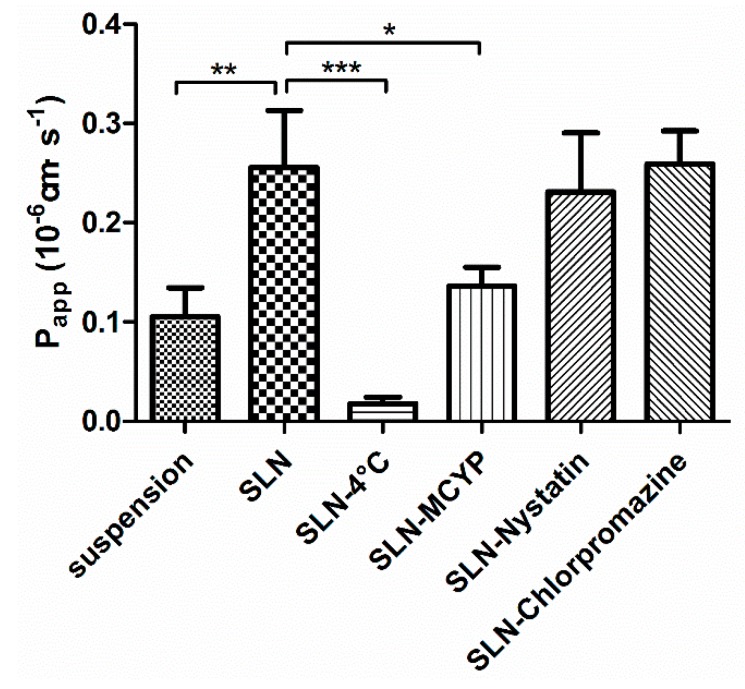
In vitro apparent permeability coefficient (*P*_app_) of CAT3 suspension and OA-CAT3-SLN after 2 h of transport across MDCK-MDR1 cell monolayer. (* *p* < 0.05, ** *p* < 0.01; *** *p* < 0.001; and *n* = 3).

The transepithelial transport of CAT3 suspension and OA-CAT3-SLN across the MDCK-MDR1 cell monolayer was determined and the results are summarized in Figure 7. CAT3 exhibited a very low *P*_app(AP-BL)_ [(0.1056 ± 0.02888) × 10^−6^ cm·s^−1^] in MDCK-MDR1 cell model at 100 ng/mL. However, the *P*_app(AP-BL)_ of OA-CAT3-SLN was (0.2557 ± 0.0578) × 10^−6^ cm·s^−1^, which was 2.42-fold higher than CAT3.

To clarify the mechanisms of enhanced drug transfer mediated by OA-CAT3-SLN, lower temperatures and transfer inhibitors were used to test the *P*_app_. When tested at 4 °C, the *P*_app(AP-BL)_ of OA-CAT3-SLN [(0.01797 ± 0.00628) × 10^−6^ cm·s^−1^] was significantly decreased (*p* < 0.05), implying that the cellular uptake OA-CAT3-SLN was energy-dependent [48]. When individually pretreated with the inhibitors chlorpromazine and nystatin, the *P*_app(AP-BL)_ of OA-CAT3-SLN was not significantly altered and demonstrated as (0.2596 ± 0.03287) × 10^−6^ cm·s^−1^ and (0.231 ± 0.05941) × 10^−6^ cm·s^−1^, respectively. However, under the influence of MCYP, the *P*_app(AP-BL)_ of OA-CAT3-SLN [(0.1362 ± 0.0188) × 10^−6^ cm·s^−1^] was significantly decreased (*p* < 0.05). Therefore, the endocytosis of OA-CAT3-SLN was not a clathrin or caveolin dependent transport but was dependent on cholesterol [49].

#### 3.3.2. In Vitro Cellular Uptake Study

**Figure 8:**
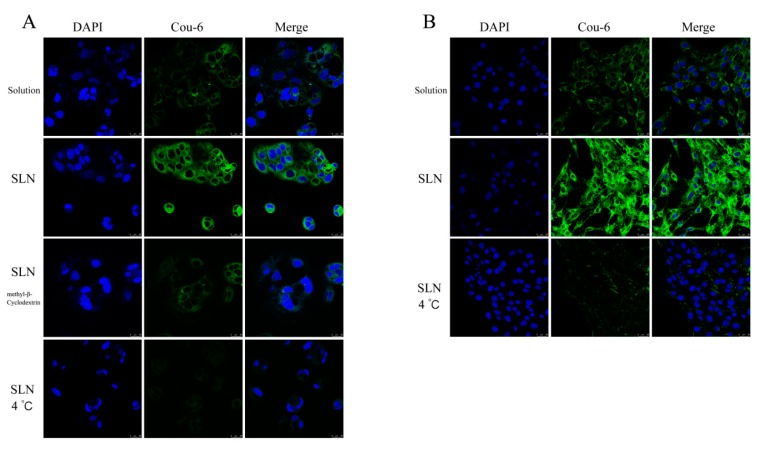
Confocal laser-scanning microscopy of internalization of fluorescent coumarin 6 (Cou-6, green) in Caco-2 (**A**) and C6 (**B**) cells after 1 h of incubation with Cou-6 suspension or Cou-6 loaded SLN (100 ng Cou-6 equivalent/mL). Cell nuclei were stained with 4′,6-diamidino-2-phenylindole (DAPI) (blue). (Scale bar: 25 μm).

The in vitro cellular uptake profile of Cou-6 loaded SLN was qualitatively analyzed by using LSCM. As shown in Figure 8A, the fluorescence intensity of Caco-2 cells incubated with Cou-6 loaded SLN was stronger than that of Cou-6 solution. However, the uptake of Cou-6 loaded SLN was significantly decreased when incubated with the inhibitor MCYP and that of at 4 °C, indicating that the cellular uptake by SLN was a cholesterol- and energy-dependent process. Similar results were observed in C6 cells (Figure 8B).

#### 3.3.3. In Vitro Cytotoxicity Analysis

**Figure 9:**
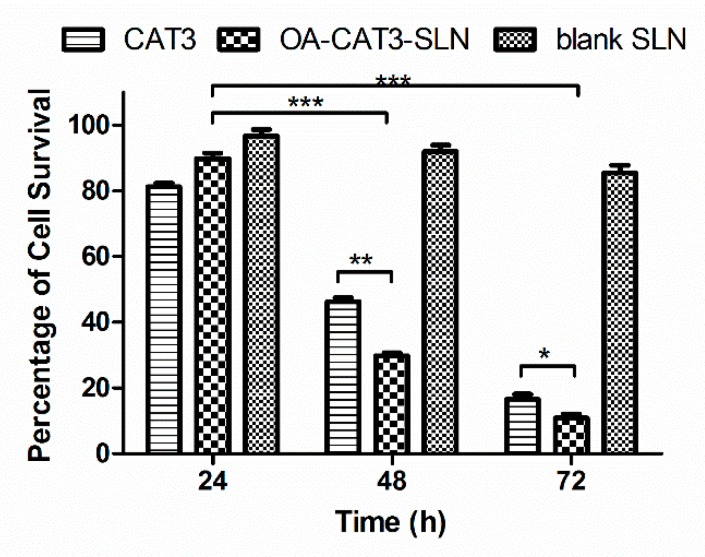
In vitro cytotoxicity evaluation of CAT3 suspension, OA-CAT3-SLN, and blank SLN in C6 glioma cells. (*p < 0.05, ** *p* < 0.01; *** *p* < 0.001; and *n* = 3).

To compare the cytotoxic differences among the CAT3 suspension, OA-CAT3-SLN, and blank SLN against the C6 glioma cell line, the in vitro cytotoxicity activity was investigated using the MTT assay. Figure 9 shows the cell viability following 24, 48, and 72 h of incubation. The results showed that the blank SLN failed to exhibit cytotoxicity in the C6 cells, and the viability was 96.58% ± 4.81%, 91.85% ± 5.00%, and 85.40% ± 5.53% for 24, 48, and 72 h, respectively, indicating the composition used to formulate SLN was biocompatible and safe in vivo [50]. Furthermore, CAT3 suspension and OA-CAT3-SLN exhibited time-dependent cytotoxicity, implying that the cell viability decreased with a long duration of contact [12]. The viability of OA-CAT3-SLN was 89.68% ± 4.33%, 29.77% ± 2.13%, and 10.75% ± 3.12% for 24, 48, and 72 h, respectively, while the cell viabilities for the CAT3 suspension were 81.15% ± 2.73%, 46.20% ± 3.12%, and 16.53% ± 3.95%, respectively. Hence, after 48 and 72 h of incubation, OA-CAT3-SLN exhibited a stronger in vitro anti-glioma efficacy than the CAT3 suspension.

#### 3.3.4. In Vivo Pharmacokinetic Studies

In the in vivo pharmacokinetic study, a simple and sensitive validated LC–MS/MS analytical method was used to determine the levels of CAT3 and its active metabolite PF403 in rat plasma, with CAT as the internal standard. Calibration curve in plasma spiked with varying concentrations of CAT3 and PF403 were linear over the concentration range of 0.14–100 ng/mL, with a determination coefficient > 0.99. The lower limit of quantification was 0.14 ng/mL. Intra- and inter-day variability (RSD) of CAT3 and PF403 in plasma were less than 15%, and the accuracy was between 95% and 120%.

**Figure 10:**
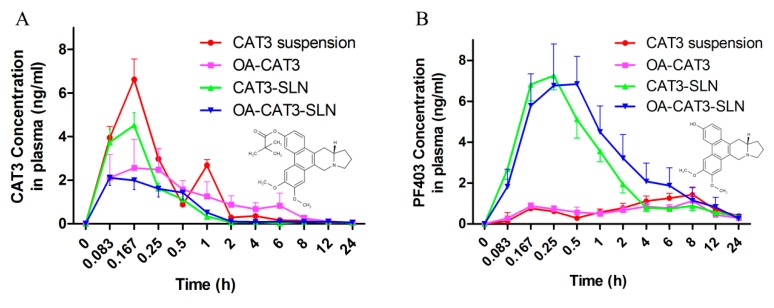
The variation of plasma concentration of CAT3 as the pro-drug form (**A**) and PF403 as the metabolite (**B**) in two animal groups as a function of time, after single oral dose administration of CAT3 suspension, OA-CAT3, CAT3-SLN, and OA-CAT3-SLN (*n* = 6).

The plasma drug concentration versus time curves after the oral administration of CAT3 suspension, OA-CAT3, CAT3-SLN, and OA-CAT3-SLN are shown in Figure 10A (CAT3, as prodrug form) and 10B (PF403, as active metabolite form), and the key pharmacokinetic parameters in plasma are listed in Table 2 and Table 3.

In Figure 10A, a double absorption peak of CAT3, as the pro-drug form in the plasma, was observed after the administration of CAT3 suspension, and the AUC_0~*t*_ was 6.39 ± 0.645 ng·mL^−1^·h. In addition, the AUC_0~*t*_ of CAT3 after administering OA-CAT3-SLN was 3.284 ± 1.254 ng·mL^−1^·h, which was less in plasma than in the CAT3 suspension, implying the presence of greater CAT3 pro-drug transfer to its active metabolite. The AUC_0~*t*_ of PF403, the metabolite form in plasma, following treatment with OA-CAT3-SLN (32.045 ± 7.425 ng·mL^−1^·h) was 1.7-fold higher than that following treatment with the CAT3 suspension (18.778 ± 1.953 ng·mL^−1^·h). Similar to the trend observed for AUC_0~*t*_ of PF403, the *C*_max_ of PF403 of OA-CAT3-SLN (7.36 ± 1.694 ng/mL) was 5.5-fold higher than in those treated with the CAT3 suspension (1.429 ± 0.171 ng/mL). Moreover, OA-CAT3-SLN significantly increased the AUC_0~*t*_ of PF403, compared with OA-CAT3 (13.713 ± 5.616) and CAT3-SLN (21.723 ± 6.763) groups, which shows that both OA-CAT3 and SLN have important roles in promoting absorption. Since the in vitro antitumor activity of PF403 is about 1000 times greater than that the pro-drug CAT3 and PF403 can penetrate the blood-brain barrier and incur an anti-glioma effects [12], the increased bioavailability and *C*_max_ of PF403, that were obtained through the oral administration OA-CAT3-SLN, would significantly contribute in exerting a better anti-glioma effect.

## 4. Discussion

Compritol 888 ATO is a natural glyceryl behenate with a number of benefits and usually used as a control release agent, or to improve the heat stability of emulsions [51,52,53]. Since the chemical stability between CAT3 and Compritol 888 ATO was superior, it was selected for formulation development. Furthermore, for the melting point of Compritol 888 ATO is not extremely high (69–74 °C), so the optimized particle size of OA-CAT3-SLN and CAT3-SLN were only about 150 nm [54].

The blank SLN, OA-CAT3-SLN, and CAT3-SLN all showed negative zeta potential values due to the carboxyl structure in the OA molecule. However, the OA-CAT3-SLN and CAT3-SLN showed a relatively slightly negative surface charge, which may be attributed to the tertiary nitrogen structure in the CAT3 molecule resisting the negative charge. Furthermore, the OA-CAT3-SLN demonstrated lower zeta potential values than the CAT3-SLN, which may indicate that OA-CAT3 has a higher solubility and was mainly in the lipid core of OA-CAT3-SLN that has less effects on the zeta potential. However, the free CAT3 in the CAT3-SLN was more likely absorbed on the lipid core surface and strongly blocked the negative charge. Hence, the OA-CAT3-SLN will have better physical stability than CAT3-SLN since it has a higher surface charge to prevent aggregation [55]. The surfactants (Tween 80 and Poloxamer 188), which are neutrally charged, were adsorbed on the particle surface and had a weak influence on zeta potential value.

The DSC results show that the melting endotherm of the lipid core in blank SLN, OA-CAT3-SLN, and CAT3-SLN was reduced from 40–75 °C compared to the melting point of Compritol 888 ATO (69–74 °C), implying that the OA can prolong the drug release from SLN [56]. Furthermore, the reduced melting points proved that the perfect crystal lattice of the Compritol 888 ATO lipid core was altered when OA was incorporated [25]. Hence, the SLN which contains OA would have higher accommodation capacity for CAT3. Moreover, there were differences in the maximum DL that was loaded on the OA-CAT3-SLN and CAT3-SLN.

A reduction in the melting point of CAT3 in the physical mixture samples of blank SLN and the CAT3 was observed in the DSC thermograms compared to that of pure CAT3. This was because the DSC results were obtained during the warming up of the environment. While the temperature was being increased, CAT3 would partly have been dissolved in the melting lipids, decreasing the melting points.

Both the PXRD and DSC results for OA-CAT3-SLN and CAT3-SLN indicated that CAT3 was present in crystalline form in CAT3-SLN, but in amorphous form in OA-CAT3-SLN. The possible reason for this phenomenon could be attributed to CAT3 demonstrating higher solubility in lipids after forming OA-CAT3, and due to the stable presentation in the lipid core, ensuring less precipitate during the cooling process of the OA-CAT3-SLN formulation. However, during the cooling process of the CAT3-SLN, the free drugs cannot acquire enough solubility in lipids if not conjugated to OA, indicating that the oversaturated drugs in aqueous phase will precipitate out and crystallize, getting absorbed on the lipid surface owing to the hydrophobic nature [57]. The inconsistent location of CAT3 in the lipid core of OA-CAT3-SLN and CAT3-SLN resulted in the differences in the in vitro drug release and EE. Lower burst drug release and higher EE were observed with OA-CAT3-SLN than with CAT3-SLN.

The CAT (Figure 1C), which is isolated from the roots of *Tylophora atrofolliculata* and *Tylophora ovate* [58], was selected as the best ISTD to test the concentration of CAT3 and PF403 in the plasma owing to its similar molecular structure and comparable pKa with CAT3 and PF403, and as it would not generate in plasma either from CAT3 nor PF403.

Since PF403 was the substrate of *P*-gp and had poor bioavailability, it could not have reached sufficiently high plasma concentrations and enter into the brain tissue to exhibit anti-glioma effects in vivo after oral administration [12,58]. Through the use of OA-CAT3-SLN, the in vitro cellular uptake and transepithelial transport were increased significantly, promoting more CAT3 conversion into PF403 in plasma, and higher bioavailability and *C*_max_ of PF403, which allowed a higher drug concentration to accumulate in the brain tissue to demonstrate enhanced anti-glioma effect.

## 5. Conclusions

The novel OA-CAT3 conjugate that has been synthesized for the first time, increased the solubility of CAT3 in lipids and was used to constructed OA-CAT3-SLN, which encapsulated amorphous drugs and had higher EE. OA-CAT3-SLN was more easily taken up by the intestinal epithelial cells in an energy-dependent cholesterol-mediated manner, resulting in higher monolayer membrane permeability. In vivo pharmacokinetic results showed that OA-CAT3-SLN can greatly increase the bioavailability and *C*_max_ of its active metabolites in plasma, which could promote the anti-glioma effects in vivo. More studies will be tested in the future within orthotopic glioma models to test the anti-tumor effects of OA-CAT3-SLN.

## Figures and Tables

**Table 1:** Physico-chemical characterization of blank SLN, OA-CAT3-SLN, and CAT3 loaded SLN (CAT3-SLN). (*n* = 3).

Formulation	Particle Size (nm)	PDI	Zeta Potential (mV)	DL (%)	EE (%)
Blank SLN	139.2 ± 2.47	0.257 ± 0.003	−41.7 ± 0.80 **	/	/
OA-CAT3-SLN	151.3 ± 17.51	0.230 ± 0.018	−26.7 ± 0.46	5.478 ± 0.346	80.65 ± 6.79
CAT3-SLN	155.7 ± 3.03	0.228 ± 0.007	−7.9 ± 0.15 **	4.067 ± 0.163 **	58.48 ± 3.35 **

** *p* < 0.01 versus OA-CAT3-SLN.

**Table 2:** The pharmacokinetic parameters of CAT3 (as the pro-drug form) in rats after oral administration (*n* = 6).

Parameters		CAT3	OA-CAT3	CAT3-SLN	OA-CAT3-SLN
AUC_(0–*t*)_	ng·mL^−1^·h	6.39 ± 0.645 **	8.632 ± 3.843 *	3.093 ± 0.133	3.284 ± 1.254
AUC_(0–∞)_	ng·mL^−1^·h	7.23 ± 0.476 **	8.954 ± 3.716 *	3.807 ± 0.441	4.356 ± 1.562
MRT_(0–*t*)_	H	3.887 ± 0.805	5.113 ± 0.616	5.433 ± 0.82	6.158 ± 0.704
MRT_(0–∞)_	H	8.779 ± 6.346	6.48 ± 2.444	13.954 ± 6.912	16.604 ± 2.597
*t*_1/2z_	H	10.824 ± 6.769	4.754 ± 3.951	16.742 ± 11.915	16.902 ± 1.578
*T*_max_	H	0.167 ± 0	0.25 ± 0.129	0.153 ± 0.034	0.125 ± 0.07
Vz/F	L/kg	21,169.855 ± 12,087.293	8806.657 ± 8572.706	62,090.614 ± 42,268.807	61,307.763 ± 18,523.656
CLz/F	L/h/kg	1388.185 ± 91.525	1268.605 ± 450.993	2653.874 ± 278.551	2576.094 ± 1027.274
*C*_max_	ng/mL	6.618 ± 0.942 **	2.711 ± 1.201	4.539 ± 1.406 **	2.245 ± 0.951

* *p* < 0.05, ** *p* < 0.01 versus OA-CAT3-SLN.

**Table 3:** The pharmacokinetic parameters of PF403 (as the metabolite) in rats after oral administration (*n* = 6).

Parameters		CAT3	OA-CAT3	CAT3-SLN	OA-CAT3-SLN
AUC_(0–*t*)_	ng·mL^−1^·h	18.778 ± 1.953 *	13.713 ± 5.616 *	21.723 ± 6.763 *	32.045 ± 7.425
AUC_(0–∞)_	ng·mL^−1^·h	24.42 ± 2.435 *	30.861 ± 24.886	25.541 ± 9.898 *	34.73 ± 6.178
MRT_(0–*t*)_	H	9.775 ± 0.104	9.677 ± 1.252	6.649 ± 1.068	5.945 ± 0.889
MRT_(0–∞)_	H	16.449 ± 0.87	56.077 ± 75.093	10.837 ± 4.978	7.742 ± 1.978
*t*_1/2z_	H	10.121 ± 0.759	39.049 ± 53.163	8.94 ± 3.181	6.218 ± 1.528
*T*_max_	H	8 ± 0	4.736 ± 3.834	0.264 ± 0.123	0.417 ± 0.129
Vz/F	L/kg	6011.79 ± 535.387	15,412.451 ± 13,804.038	5298.387 ± 1424.022	2807.484 ± 748.904
CLz/F	L/h/kg	412.919 ± 41.283	442.039 ± 193.353	439.687 ± 152.452	333.068 ± 131.27
*C*_max_	ng/mL	1.429 ± 0.171 **	1.431 ± 0.315 **	7.884 ± 1.621	7.36 ± 1.694

* *p* < 0.05, ** *p* < 0.01 versus OA-CAT3-SLN.

## Abbreviations

CAT3	13a-(S)-3-pivaloyloxyl-6,7-dimethoxyphenanthro(9,10-b)-indolizidine
PF403	13a-(S)-3-hydroxyl-6,7-dimethoxyphenanthro(9,10-b)-indolizidine
GBM	Glioblastoma multiforme
TMZ	Temozolomide
SLN	Solid lipid nanoparticles
MDCK-MDR1	Madin-Darby canine kidney cells transfected with the human MDR1 gene
SD	Sprague-Dawley
NMR	Nuclear magnetic resonance
TMS	Tetramethylsilane
DL	Drug loading
EE	Encapsulation efficiency
OA	Oleic acid
OA-CAT3	Oleic acid and CAT3 conjugate
OA-CAT3-SLN	OA-CAT3 loaded SLN
CAT3-SLN	With the same composition and contents to the OA-CAT3-SLN except the preparation of OA-CAT3 in advance
DSC	Differential scanning calorimetry
FTIR	Fourier transform infrared spectroscopy
API	Active pharmaceutical ingredient
PVDF	Polyvinylidene fluoride
TEER	Transepithelial electrical resistance
DI	Deionized
PDI	Polydispersity index
HBSS	Hanks’ balanced salt solution
AP	Apical side
BL	Basolateral
LC-MS/MS	Liquid chromatography-mass spectrometry
*P*_app_	Apparent permeability coefficient
HPLC	High-performance liquid chromatography
C6-luc	Luciferase-expressing C6 cells
DAPI	4′,6-diamidino-2-phenylindole
LSCM	laser scanning confocal microscopy
MTT	3-(4, 5-dimethylthiazol-2-yl)-2,5 diphenyltetrazolium bromide
MCYP	Methyl-β-Cyclodextrin
PBS	Phosphate-buffered saline
Cou-6	Fluorescent Coumarin 6
ISTD	Internal standard
DAS	Drug and Statistics
